# The Oceanian archaeological and palaeontological isotope database

**DOI:** 10.1016/j.dib.2026.112722

**Published:** 2026-03-31

**Authors:** S. Anna Florin, Ashleigh J. Rogers, Natasha B. Lyall, Alison Crowther, Carlo Cocozza, Ricardo Fernandes

**Affiliations:** a School of Archaeology and Anthropology, The Australian National University, Canberra, ACT 2601, Australia; b Centre of Excellence for Indigenous Environmental Histories and Futures, The Australian National University, Canberra, ACT 2601, Australia; c Monash Indigenous Studies Centre, Monash University, Melbourne, VIC 3800, Australia; d Centre of Excellence for Indigenous Environmental Histories and Futures, Monash University, Clayton, VIC 3800, Australia; e School of Social Science, The University of Queensland, Brisbane, QLD 4072, Australia; f Department of Archaeology, Max Planck Institute of Geoanthropology, Kahlaische Str. 10, Jena 07745, Germany; g ArchaeoBioCenter (ABC), Ludwig-Maximilians-Universität München, Geschwister-Scholl-Platz 1, München, 80539, Germany; h Dipartimento di Scienze e Tecnologie Ambientali Biologiche e Farmaceutiche (DiSTABiF) and Mediterranean bioArchaeological Research Advances (MAReA) centre, Università degli studi della Campania “Luigi Vanvitelli”, Via Vivaldi 43, Caserta, 81100, Italy; i Faculty of Archaeology, University of Warsaw, Krakowskie Przedmieście 26/28, Warsaw 00-927, Poland; j Climate Change and History Research Initiative, Princeton University, Princeton, NJ 08544, USA

**Keywords:** Isotopes, Archaeology, Palaeontology, Pacific, Australia, Foodways, Palaeoenvironment

## Abstract

The Oceanian Archaeological and Palaeontological Isotope Database (OAPID) is a comprehensive open-access dataset of isotopic measurements derived from Pleistocene and Holocene archaeological and palaeontological sites in the Oceanian region, including within Australasia, Melanesia, Micronesia, and Polynesia. OAPID encompasses carbon, nitrogen, oxygen, strontium and sulphur isotope measurements from organic, organic phosphate, and carbonate fractions of archaeologically and paleontologically derived human, animal and plant remains, as well as extensive supporting information regarding their geographic, temporal and cultural context. With 6465 individual entries spanning more than four decades of isotopic research in Oceania, this resource will facilitate ongoing research into palaeoenvironments, past lifeways and subsistence systems, and present management of animals and vegetation.

Specifications Table

**Table:** 

Subject	Social Sciences
Specific subject area	Archaeology; palaeontology
Type of data	Secondary
Data collection	Data was located using the online scientific search engine Google Scholar. Original publications and compilations containing carbon, nitrogen, oxygen, strontium and sulphur isotope measurements on human, non-human animals or plants from archaeological or palaeontological sites in Oceania were located. From these we extracted metadata information concerning the site, sample, context, chronology and isotopic measurements. Literature published up to 1 Jan 2025 is included in the database.
Data source location	All sources of raw data are identified in the dataset.
Data accessibility	Repository name: Pandora Data identification number: https://www.doi.org/10.48493/9esb-cq90 Direct URL to data: https://pandoradata.earth/dataset/the-oceanian-archaeological-and-palaeontological-isotope-database
Related research article	A.J. Rogers, S.A. Florin, A. Crowther, N.B. Lyall, C. Cocozza, R. Fernandes, A meta-analysis of stable isotope data from early Pacific Island colonisation to complex chiefdoms, OSF Preprint (2026). https://doi.org/10.31235/osf.io/b2hwg_v1.

## 1 Value of the Data

• OAPID is a research tool designed for archaeologists, palaeoanthropologists, palaeontologists and palaeoecologists employing isotopic data.
• Isotopic data compiled in OAPID can be used to investigate past trends in human diet, trade and social hierarchy, human and animal movements, and animal management, as well as to reconstruct past climates and environments in the Oceanian region at various spatiotemporal scales of resolution.
• OAPID can be used to identify and fill research gaps in isotopic studies on the region.

## 2 Background

**Fig. 1:**
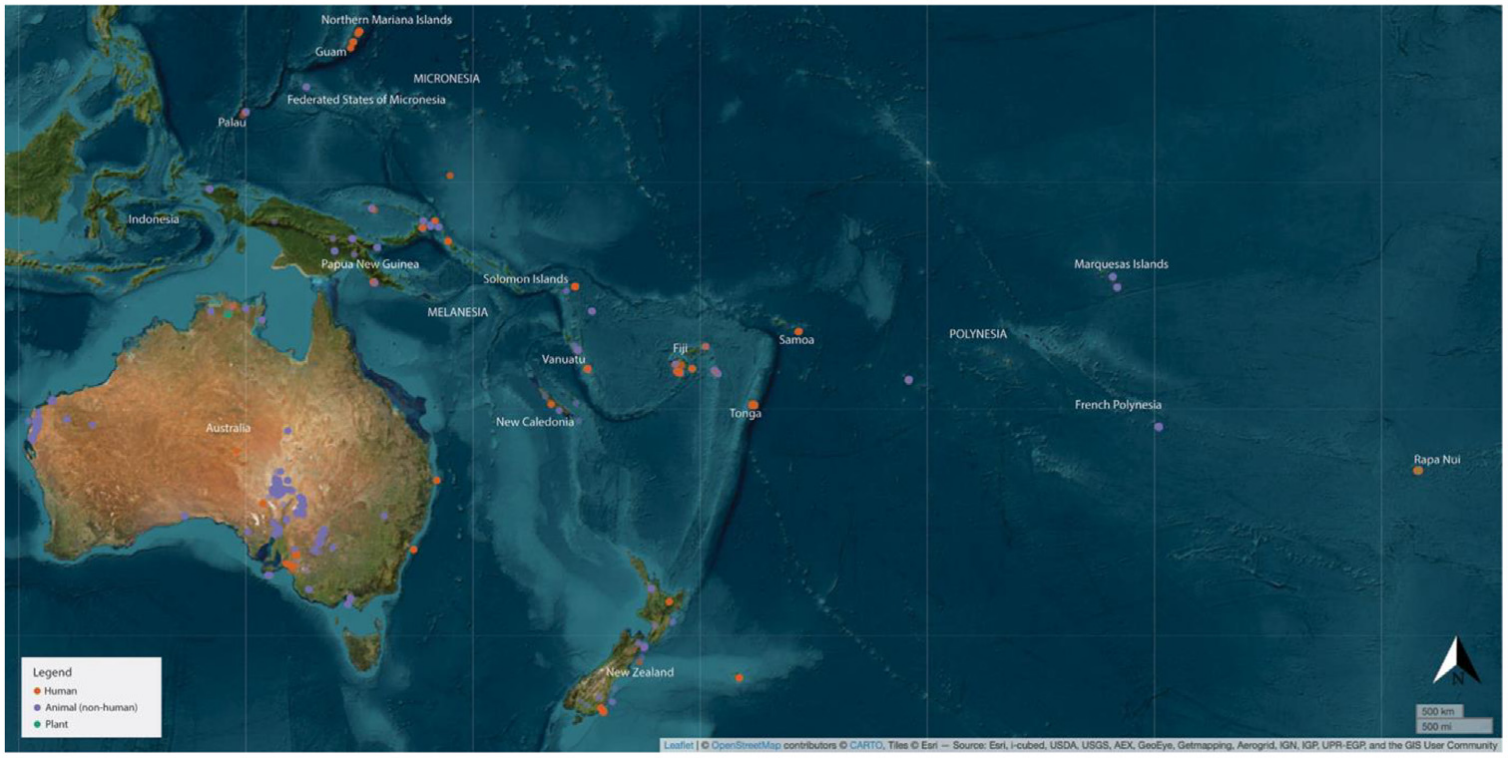
Map of Oceania, illustrating the spread and type of palaeo-isotopic data compiled in OAPID.

Oceania incorporates a vast area of land and sea, with >10,000 islands, ranging in size from continental landmasses (Australia) to uplifted coral atolls, scattered across the Pacific Ocean between the continents of Asia and the Americas (see Fig. 1). It is host to diverse environments, incorporating tropical, temperate and arid flora and fauna, and to equally diverse but interconnected human histories, dating from 65,000 years ago in Australia and New Guinea [1], 42,000 years ago in the western Pacific [2], and c. 3000–700 years ago in the central, eastern and southern Pacific [[3], [4], [5], [20]]. More recent European exploration and settlement has led to further sociocultural and environmental transformations with wide-ranging impacts on Indigenous communities today.

Isotopic research has the capability to elucidate our understandings of past lifeways, including past diets, trade and environments. OAPID has been designed to compile carbon, nitrogen, oxygen, strontium and sulphur isotope measurements from human, animal and plant remains from across Oceania with the aim of allowing for large-scale investigations of spatiotemporal isotopic variations across this disparate but inter-connected region.

## 3 Data Description

OAPID is accessible via the Pandora data platform (https://pandoradata.earth/the-oceanian-archaeological-and-palaeontological-isotope-database) within The Oceanian Archaeological and Palaeontological Isotope Database Repository for datasets at: https://www.doi.org/10.48493/9esb-cq90. OAPID is part of the IsoMemo network (https://isomemo.com/), a collaborative open-access initiative of independent isotopic databases. In line with the ethos of the initiative, the database adheres to FAIR principles for correct open data management practices.

OAPID is composed of two resources. 1) A text file (.pdf) listing all original data sources from where isotopic data was retrieved; 2) A spreadsheet file, available both in .xlsx (Excel) and .csv (Comma Separated Value) formats, storing the collected isotopic data. The spreadsheet hosts 6465 entries, including 2169 isotopic measurements on human, 4136 on animal and 160 on plant remains recovered from archaeological and palaeontological contexts from the Oceania region. Table 1 provides the list of fields and their descriptors for OAPID. A description follows below.

**Table 1:** Protocol for database fields. ‘Category’ refers to the relevance of a database field to specific categories of isotopic data, i.e. relevant to all categories (All) or relevant only to measurements taken from human (H), non-human animal (A), or plant (P) remains, or any combination of the above.

Field	Category	Input type	Input
Entry ID	All	Numerical field (consecutive)	A unique identifier code for the data entry.
Submitter ID	All	Formatted text field	Last name and initials of the person submitting the data entry (e.g., Florin, S.A., or Rogers, A.J.).
Submission Date	All	Date field (‘dd/mm/yy’)	Date of data entry.
Comments	All	Free form text field	Any relevant comments about the data entry.
Sample ID	All	Free form text field	ID of sample as reported in the publication.
Individual ID	All	Free form text field	ID of individual as reported in the publication. Individual ID may be different to sample ID where more than one sample has been analysed from the same individual or where there is a single sample from an individual but a different sample code was used for isotopic analysis.
Entry category	All	Categorical field	Report broad taxonomic type as ‘Human’, ‘Animal (non-human)’, or ‘Plant’ to enable quick data filtering.
Higher taxonomic classification	All	Formatted text field	Taxonomic classifications higher than Family level (Kingdom, Phylum, Class, Order). To be used for samples that cannot be identified to Family or lower classifications (e.g., Aves). Where Family or lower classifications have been recorded put ‘NA’ (not applicable).
Family	All	Formatted text field	Taxonomic family of individual (e.g., Suidae). Enter ‘NR’ if not reported. In this instance, taxonomic information should be present in the ‘Higher taxonomic classification’ field.
Genus	All	Formatted text field	Taxonomic genus of individual (e.g., *Sus*). Enter ‘NR’ if not reported.
Species and subspecies	All	Formatted text field	Taxonomic species and subspecies of individual (e.g., *scrofa domesticus*). Enter ‘NR’ if not reported.
Taxonomy descriptive category	All	Free form text field	Include additional descriptive information relative to taxonomic identification (e.g., fruiting, marine mammal, large bovid). Where there is no relevant information put ‘NA’ not applicable.
Common name	All	Formatted text field	Common English name of individual (e.g., pig, coconut).
Extinct	All	Categorical field	Is the identified taxonomic group extinct (i.e., are there no living members)? Report ‘Y’ (yes), ‘N’ (no).
Wild or domesticated	A, P	Categorical field	Is the animal/plant considered to be wild or domesticated? Report ‘W’ (wild), ‘D’ (domesticated), or ‘U’ (unable to determine; e.g., Aves could be chicken or seabird). Enter ‘NA’ (not applicable) for humans.
Living environment	A, P	Categorical field	Living environment of individual, including ‘Brackish’, ‘Freshwater’, ‘Marine’ and ‘Terrestrial’. Enter ‘NA’ (not applicable) for humans.
Trophic behaviour	All	Categorical field	Trophic behaviour of individual, including ‘Carnivore’, ‘Detritivore (filter-feeder)’, ‘Herbivore’, ‘Omnivore’ and ‘Primary producer’.
Native or introduced	A, P	Categorical field	Is the individual native or introduced? ‘Native’ (native), ‘Introduced” (introduced), or ‘U’ (unable to determine; e.g., if higher order classification like Aves and could be chicken or seabird). Enter ‘NA’ (not applicable) for humans.
Biological sex	H, A	Categorical field	Sex of individual, including ‘F’ (female), ‘M’ (male), ‘F?’ (likely female), ‘M?’ (likely male), ‘I’ (impossible to determine; e.g., juveniles), ‘U’ (unable to determine due to preservation), or ‘NR’ (not reported). Enter ‘NA’ (not applicable) for plants.
Reported age	H, A	Free form text field	Age of individual, written as reported in the publication. Enter ‘NR’ (not reported) when information is absent, and ‘NA’ (not applicable) for plants.
Human age class	H	Categorical field	Age class of individual assigned using Buikstra and Ubelaker’s (1994) age classes, as follows: ‘Fetus’ (before birth), ‘Infant’ (0–3 years), ‘Young juvenile’ (3–7 years), ‘Old juvenile’ (7–13 years), ‘Adolescent’ (13–18 years), ‘Young adult’ (18–26 years), ‘Young middle adult’ (26–36 years), ‘Old middle adult’ (36–46 years), ‘Mature adult’ (46+ years). Where there is only a lower age limit given, use: ‘Adult’ (lower age limit between 18 and 30 years), ‘Mature adult’ (lower age limit greater than 46 years). Where age ranges given do not fit classifications precisely (e.g., 16–32), report all age classes separated by a semicolon. Where publications use categories (e.g., “child”, “young adult”) without any mention of their numerical equivalency or system of classification use ‘Sub-adult’ (0–18 years and ‘Adult’ (18–80). Use ‘U’ (unable to determine due to preservation) and ‘NR’ (not reported) as appropriate for humans. Enter ‘NA’ (not applicable) for animals and plants.
Min. age individual (years)	H	Numerical field	Minimum age reported. If no numeric age is reported, this refers to the broad age class that is reported in the original publication. N.B., if no age is specified, a ‘0′ is registered.
Max. age individual (years)	H	Numerical field	Maximum age reported. If no numeric age is reported, this refers to the broad age class that is reported in the original publication. N.B., if no age is specified, an ‘80′ is registered.
Increment analysis	H, A	Categorical field	If the sample is analysed in increments to represent different ages through time. Applied to studies of teeth and hair. Enter ‘Y’ (yes) or ‘N’ (no) for tooth or hair samples and ‘NA’ (not applicable) for all other sample types (e.g., bone, mollusc shell, plants).
Increment reported age	H, A	Free form text field	The age reported for the sampled increment. ‘NA’ (not applicable) where increment analysis was not performed.
Probable human cultural group	H	Categorical field	Most likely regional cultural grouping of the individual, including ‘Aboriginal and Torres Strait Islander’, ‘East Polynesian’, ‘European’, ‘Lapita’, ‘Makassan’, ‘Melanesian’, ‘Micronesian’, ‘West Polynesian’. ‘NA’ (not applicable) for animals and plants.
Sampled element	All	Categorical field	This identifies what was sampled for isotopic analysis. Includes ‘Bone’, ‘Tooth’, ‘Seed’, ‘Endocarp’, ‘Wood’, ‘Shell’, ‘Eggshell’, ‘Leaf’.
Bone type	H, A	Categorical field	Reports bone type analysed (e.g., ‘femur’, ‘humerus’, ‘rib’, ‘long bone’). If bone type is not reported enter ‘NR’ (not reported). If sampled element was not a bone, enter ‘NA’ (not applicable).
Bone side	H, A	Categorical field	If a bone is analysed, this category reports the body side as ‘L’ (left), ‘R’ (right), or ‘NR’ (not reported), or ‘NA’ (not applicable; i.e., not a paired element e.g., vertebrae). If sampled element was not a bone, also report ‘NA’ (not applicable).
Tooth type as reported	H, A	Free form text field	Tooth type (e.g., molar, premolar) as reported in the paper.
Tooth set	H, A	Categorical field	Tooth is ‘Permanent’, ‘Deciduous’, or ‘NR’. Enter ‘NA’ (not applicable) where sampled element is not a tooth.
Tooth position	H, A	Categorical field	Position of tooth in mandible or maxilla. Report as ‘Mandibular’, ‘Maxillary’, or ‘NR’ (not reported). Enter ‘NA’ (not applicable) where sampled element is not a tooth.
Tooth side	H, A	Categorical field	If a tooth is analysed, this category reports the body side as ‘L’ (left), ‘R’ (right), or ‘NR’ (not reported). If sampled element was not a tooth, enter ‘NA’ (not applicable).
Tooth type	H, A	Categorical field	Identified tooth type and number (e.g., I1, I2, C, PM1, PM2, M1, M2, M3). If the tooth number is unknown record as ‘Incisor’, ‘Canine’, ‘Premolar’, ‘Molar’. If sampled element was not a tooth, enter ‘NA’ (not applicable).
Plant sample status	P	Categorical field	Archaeobotanical preservation, including ‘Charred’, ‘Biomineralised’, or ‘Dried’. Enter ‘NA’ (not applicable) for humans and animals.
Analysed component	H, A	Categorical field	Component analysed in the following categories: ‘Bone collagen’, ‘Bone bioapatite, ‘Dentine collagen’, ‘Dentine mineral’, ‘Tooth Enamel’. In cases where different components are analysed from the same sample, report all components separated by a semicolon (e.g., Bone collagen; Bone bioapatite).
Site name	All	Free form text field	Site name as reported in the publication. If from a museum collection, enter ‘NR’ (not reported).
Region	All	Free form text field	Province, state, territory, or island the site is located within. Use ‘NR’ (not reported) if required.
Country	All	Free form text field	Country the site is located within.
Year excavated	All	Date field (‘yyyy’, ‘yyyy; yyyy’ or ‘yyyy-yyyy’)	Date(s) of excavation. Use a dash where excavation years are consecutive and a semicolon when there are multiple non-consecutive excavation years. Enter ‘NR’ (not reported) when the year the samples were excavated is not reported.
Altitude (m asl)	All	Numerical field	Altitude of site recorded in metres above sea level. For unreported query Google Elevation API.
Latitude	All	Numerical field	Latitude of site recorded in decimal degrees.
Longitude	All	Numerical field	Longitude of site recorded in decimal degrees.
Exact site location	All	Categorical field	Are the exact site location coordinates reported? Enter ‘Y’ (yes) or ‘N’ (no).
Radius	All	Numerical field	Approximate radius within which the site is contained from the location data. Enter in kilometres. Where the exact site location coordinates are reported, report as ‘0′.
Site description	All	Free form text field	Site description as reported in the publication. When description is absent enter ‘NR’ (not reported).
Site type	All	Categorical field	Site type, including ‘architectural’, ‘lake’, ‘open site’, ‘cave/rockshelter’, and ‘maritime’. When site type information is absent enter ‘NR’ (not reported).
Context description	All	Free form text field	Context description as reported in the publication, including description of any associated grave goods if context is a burial. When description is absent enter ‘NR’ (not reported).
Context ID	All	Free form text field	The ID of the context as reported in the original publication. When context ID information is absent enter ‘NR’ (not reported).
Presence of burial goods	H	Categorical field	Are burial goods reported? ‘Y’ (yes), ‘N’ (no) or ‘NR’ (not reported). If the site is not a burial, enter ‘NA’ (not applicable). Specific burial good information is reported in context description field.
Min. date	All	Numerical field	Reported using CE and BCE, where BCE dates are reported as negative numbers using a minus sign (e.g., −800). This is the 95% credible interval when Bayesian chronological modelling was employed.
Max. date	All	Numerical field	Reported using CE and BCE, where BCE dates are reported as negative numbers using a minus sign (e.g., −800). This is the 95% credible interval when Bayesian chronological modelling was employed.
Dating method	All	Categorical field	Dating method used, including ‘Historical/material culture evidence’, ‘Optically stimulated luminescence’, ‘Radiocarbon’, ‘Obsidian hydration’, ‘Uranium-thorium’, ‘Modelling’, ‘Material culture typology’, or ‘NR’ (not reported).
Date target	All	Categorical field	The broad target of the date, including ‘Site’, ‘Layer’, ‘Phase’, ‘Sample’.
Radiocarbon ID	All	Free form text field	Sample lab code for radiocarbon. Should only be reported if the date is taken from the analysed sample. If more than one date is available for the sample, the IDs are separated by semicolon. Multiple dates should be reported in the same order that they appear in under 14C (BP) and 14C unc (BP) fields. Use “NR” when not reported, and “NA” (not applicable) when inapplicable.
14C (BP)	All	Numerical field	Uncalibrated 14C date in BP. Does not include intervals. Should only be reported if the date is taken from the analysed sample. If more than one date is available for the sample, they are separated by semicolon. Multiple dates should be reported in the same order that they appear in under Radiocarbon ID and 14C unc (BP) fields.
14C unc. (BP)	All	Numerical field	14C uncertainty. Separate multiple dates by semicolon. Multiple dates should be reported in the same order that they appear in under Radiocarbon ID and 14C (BP) fields.
Local marine reservoir effect (mean)	All	Numerical field	Local marine radiocarbon reservoir effect correction (ΔR).
Local marine reservoir effect (unc.)	All	Numerical field	Local marine radiocarbon reservoir effect correction (ΔR) uncertainty.
Period tags	All	Categorical field	Regional historical period tags separated by a semicolon, including in Australia: ‘Pleistocene’, ‘Early Holocene’, ‘Middle Holocene’, ‘Late Holocene’ and ‘Historic’; in Melanesia and the Western Pacific: ‘Pleistocene’, ‘Pre-Lapita’, ‘Lapita’, ‘Post-Lapita’, ‘Proto-historic’ and ‘Historic’; in the East Pacific: ‘Early prehistoric’, ‘Middle prehistoric’ and ‘Late prehistoric’, and Micronesia: ‘Pre-Latte’, ‘Latte’, ‘Post-Latte’, and ‘Historic’. Where date ranges do not fit classifications precisely, report all applicable period tags separated by a semicolon. See additional file for period tag date ranges. These ranges are broad and Min. date and Max. date fields should be used for fine-grained regional historical period analyses.
Reference	All	Formatted text field	Bibliographical reference written in the Harvard style with all commas replaced by semicolons.
URL link	All	Formatted text field	Hyperlink to internet site where publication is available.
DOI	All	Formatted text field	DOI of publication. ‘NA’ (not applicable) where DOI does not exist.
Year of publication	All	Numerical field	Year of publication.
Compilation paper reference	All	Formatted text field	Bibliographical reference of related compilation publication written in the Harvard style with all commas replaced by semicolons.
Compilation paper URL link	All	Formatted text field	Hyperlink to internet site where compilation publication is available.
Compilation paper DOI	All	Formatted text field	DOI of compilation publication. ‘NA’ (not applicable) where DOI does not exist.
Compilation paper year of publication	All	Numerical field	Year of compilation publication.
Lab name organic δ^13^C & δ^15^N	All	Free form text field	Name of laboratory where carbon and/or nitrogen measurements were carried out.
Nr. of measurements organic δ^13^C & δ^15^N	All	Numerical field	Number of measurements on separately pre-treated samples (i.e., when more than one individual or sample is aggregated; n.b., this does not include replicate measurements done on the same pretreated material in the same lab).
Sample preparation organic δ^13^C & δ^15^N	All	Free form text field	Description of sample preparation and pretreatment for carbon and/or nitrogen measurements as reported in the publication.
Analysis organic δ^13^C & δ^15^N	All	Free form text field	Description of sample analysis parameters and instruments for carbon and/or nitrogen measurements as reported in the publication.
Organic AMS δ^13^C (VPDB)	All	Numerical field	*δ^13^C* measurement in organic samples through an AMS machine.
Organic AMS δ^13^C (VPDB) unc.	All	Numerical field	*δ^13^C* measurement uncertainty in organic samples through an AMS machine.
Organic IRMS δ^13^C (VPDB)	All	Numerical field	*δ^13^C* measurement in organic samples through an IRMS machine.
Organic IRMS δ^13^C unc. (VPDB)	All	Numerical field	*δ^13^C* measurement uncertainty in organic samples through an IRMS machine. N.B., this is a combined uncertainty typically determined from replicate measurements.
δ^15^N (AIR)	All	Numerical field	*δ^15^N* measurement.
δ^15^N unc. (AIR)	All	Numerical field	*δ^15^N* measurement uncertainty. N.B., this is a combined uncertainty typically determined from replicate measurements.
Collagen yield	H, A	Numerical field	Yield from collagen extraction in percentage.
Atomic C/N ratio	All	Numerical field	Atomic C/N ratio in sample.
%C	All	Numerical field	Elemental concentration of carbon in organic sample.
%N	All	Numerical field	Elemental concentration of nitrogen in sample.
Lab name δ34S	All	Free form text field	Name of laboratory where sulphur measurements were carried out.
Nr. of measurements δ34S	All	Numerical field	Number of measurements on separately pre-treated samples. N.B., this does not include replicate measurements done on the same pretreated material in the same lab.
Nr. of measurements δ34S	All	Free form text field	Description of sample preparation and pretreatment for sulphur measurements as reported in the publication.
Analysis δ34S	All	Free form text field	Description of sample analysis parameters and instruments for sulphur measurements as reported in the publication.
δ34S (VCDT)	All	Numerical field	*δ34S* measurement in sample reported as relative to VCDT standard.
δ34S (VCDT) unc.	All	Numerical field	Uncertainty of *δ34S* measurement in sample reported as relative to VCDT standard.
Atomic C/S ratio	All	Numerical field	Atomic C/S ratio in sample.
Atomic N/S ratio	All	Numerical field	Atomic N/S ratio in sample.
%S	All	Numerical field	Elemental concentration of sulphur in sample.
Lab name carbonate δ^13^C & δ^18^O	All	Free form text field	Name of laboratory where carbonate carbon and oxygen measurements were carried out.
Nr. of measurements carbonate δ^13^C & δ^18^O	All	Numerical field	Number of measurements on separately pre-treated samples. N.b., this does not include replicate measurements done on the same pretreated material in the same lab.
Sample preparation carbonate δ^13^C & δ^18^O	All	Free form text field	Description of sample preparation and pretreatment for carbon and oxygen measurements as reported in the publication.
Analysis carbonate δ^13^C & δ^18^O	All	Free form text field	Description of sample analysis parameters and instruments for carbon and oxygen measurements as reported in the publication.
Carbonate δ^13^C (VPDB)	All	Numerical field	*δ^13^C* measurement in carbonate.
Carbonate δ^13^C unc. (VPDB)	All	Numerical field	Uncertainty of *δ^13^C* measurement in carbonate.
Carbonate δ^18^O (VPDB)	All	Numerical field	*δ^18^O* measurement in carbonate reported as relative to VPDB standard.
Carbonate δ^18^O unc. (VPDB)	All	Numerical field	Uncertainty of *δ^18^O* measurement in carbonate reported as relative to VPDB standard.
Carbonate δ^18^O (VSMOW)	All	Numerical field	*δ^18^O* measurement in carbonate reported as relative to VSMOW standard.
Carbonate δ^18^O unc. (VSMOW)	All	Numerical field	Uncertainty of *δ^18^O* measurement in carbonate reported as relative to VSMOW standard.
Lab name phosphate δ^18^O	All	Free from text field	Name of laboratory where phosphate oxygen measurements were carried out.
Nr. of measurements phosphate δ^18^O	All	Numerical field	Number of measurements on separately pre-treated samples. N.B., this does not include replicate measurements done on the same pretreated material in the same lab.
Sample preparation phosphate δ^18^O	All	Free form text field	Description of sample preparation and pretreatment for phosphate oxygen measurements as reported in the publication.
Analysis phosphate δ^18^O	All	Free form text field	Description of sample analysis parameters and instruments for phosphate oxygen measurements as reported in the publication.
Phosphate δ^18^O (VPDB)	All	Numerical field	*δ^18^O* measurement in phosphate reported as relative to VPDB standard.
Phosphate δ^18^O unc. (VPDB)	All	Numerical field	Uncertainty of *δ^18^O* measurement in phosphate reported as relative to VPDB standard.
Phosphate δ^18^O (VSMOW):	All	Numerical field	*δ^18^O* measurement in phosphate reported as relative to VSMOW standard.
Phosphate δ^18^O unc. (VSMOW)	All	Numerical field	Uncertainty of *δ^18^O* measurement in phosphate reported as relative to VSMOW standard.
Lab name ^87^Sr/^86^Sr	All	Free form text field	Name of laboratory where strontium measurements were carried out.
Nr. of measurements ^87^Sr/^86^Sr	All	Numerical field	Number of measurements on separately pre-treated samples. N.B., this does not include replicate measurements done on the same pretreated material in the same lab.
Sample preparation ^87^Sr/^86^Sr	All	Free form text field	Description of sample preparation and pretreatment for strontium measurements as reported in the publication.
Analysis ^87^Sr/^86^Sr	All	Free text field	Description of sample analysis parameters and instruments for strontium measurements as reported in the publication.
^87^Sr/^86^Sr	All	Numerical field	*^87^Sr/^86^Sr* measurement in sample.
^87^Sr/^86^Sr unc.	All	Numerical field	Uncertainty of *^87^Sr/^86^Sr* measurement in sample.
Lab name δ34S	All	Free form text field	Name of laboratory where sulphur measurements were carried out.
Nr. of measurements δ34S	All	Numerical field	Number of measurements on separately pre-treated samples (n.b., this does not include replicate measurements done on the same pretreated material in the same lab).
Sample preparation δ34S	All	Free text field	Description of sample preparation and pretreatment for sulphur measurements as reported in the publication.
Analysis δ34S	All	Free text field	Description of sample analysis parameters and instruments for sulphur measurements as reported in the publication.
δ34S (VCDT)	All	Numerical field	δ34S measurement in sample reported as relative to VCDT standard.
δ34S (VCDT) unc.	All	Numerical field	Uncertainty of δ34S measurement in sample reported as relative to VCDT standard.
Atomic C/S ratio	All	Numerical field	Atomic C/S ratio in sample.
Atomic N/S ratio	All	Numerical field	Atomic N/S ratio in sample.
%S	All	Numerical field	elemental concentration of sulphur in sample.

Each entry into the database is associated to a unique progressive numeric ‘Entry ID’. In addition, any sample and individual specimen numbers as per the original publication are reported (‘Sample ID’, Individual ID’). Multiple fields are used to classify the samples by category and taxonomic description, including: ‘Entry category’, which reports the broad category of the sample as ‘Human’, ‘Animal (non-human)’, or ‘Plant’, enabling quick data filtering; a series of taxonomic descriptors to allow for different levels of taxonomic identification (‘Higher taxonomic classification’, ‘Family’, Genus’, ‘Species and subspecies’, ‘Taxonomy descriptive category’); information pertaining to nature and status of the taxon, such as whether the taxon is extinct or extant (‘Extinct’) and details of the taxon’s ‘Trophic behaviour’. For non-human animals and plants, further contextual information surrounding their status as ‘Wild or domesticated’, ‘Native or introduced’ and their known ‘Living environment’ is also provided.

More information pertaining to the sampled individual and element is also provided. For all categories, the ‘Sampled element’ subjected to isotopic analysis is reported. For humans and non-human animal remains, the ‘Biological sex’ and ‘Reported age’ of the individual is also recorded, in addition to details of the skeletal element analysed (‘Bone type’, ‘Bone side’, ‘Tooth type as reported’, ‘Tooth set’, ‘Tooth position’, ‘Tooth side’, ‘Tooth type’), any incremental analyses(‘Increment analysis’, ‘Reported increment age’), and the analysed osteological component (‘Analysed component’). For human remains, additional data is provided for the age and cultural background of the individual. This includes the ‘Human age class’, which follows a modified Buikstra and Ubelaker [6] standard classification (described in Table 1), and the minimum and maximum age of the individual (‘Min. age individual (years)’, ‘Max. age individual (years)’), allowing for easy data analysis across age classes. The ‘Probable cultural background’ of human remains is assigned according to the most likely regional cultural grouping of the individual, including Aboriginal and Torres Strait Islander, Melanesian, East or West Polynesian, Micronesian, Lapita, Makassan and European. For plants, the ‘Plant sample status’ or preservation status of the sample is registered (e.g., charred, desiccated, mineralised).

Locational, contextual, chronological, and reference data are provided for all samples. The ‘Site name’, ‘Region’ and ‘Country’ from which the sample was excavated as well as the year excavated, if known, is provided. The site’s ‘Altitude’, ‘Latitude’ and ‘Longitude’ (the latter two provided in decimal places) are also given, allowing for spatial analyses of the data. To allow for sites without published coordinates, the ‘Exact site location’ and ‘Radius’ fields enable for an informed approximate location to be defined using information in the original publication (e.g., map, place name). Contextual data for each site are recorded in both descriptive and categorised formats in ‘Site description’, ‘Site type’, ‘Context description’, ‘Context ID’, and, in the case of human remains, ‘Presence of burial goods’ fields. Chronological data provided includes data on the minimum and maximum age estimates for the sample reported as CE and BCE (‘Min. date’, ‘Max. date’), ‘Dating method’, and the target of the dating analysis (‘Date target’). Dates are reported in the database as numerical categories, therefore whenever a BCE date was registered, a minus sign (-) was added before the date to flag this. In the case of radiocarbon dating, the ‘Radiocarbon ID’, the uncalibrated radiocarbon age (14C (BP)), and its uncertainty (14C unc. (BP)), and, where applicable, the ‘Local marine reservoir effect (mean)’ and its uncertainty (Local marine reservoir effect (unc.)) are also provided. In order to allow for analysis using regionally and culturally significant temporal categories, a further field denoting ‘Period tags’ is used. Table 2 lists the different period tags used in the OAPID database. Reference information, including the ‘Reference’ formatted in Harvard style, ‘URL link’, unique digital identifier (‘DOI’), and ‘Year of publication’ is provided for the publication and/or compilation publication from which the isotopic data has been compiled.

**Table 2:** Regional and chronological data for period tags used in OAPID. Note that these date ranges are intentionally broad and may not be exact for specific island groups due to the stepwise nature of Pacific Island colonisation.

Geographic Region	Period Tag	Date Range (BP)	Date Range (BCE/CE)	Reference
**Australia**	Pleistocene	Pre-11,700	Pre-9750 BCE	NA
	Early Holocene	11,700–8150	9750–6200 BCE	NA
	Mid-Holocene	8150–4950	6200–3000 BCE	NA
	Late Holocene	4950–162	3000 BCE–1788 CE	NA
	Historic (contact)	Post-162	Post-1788 CE	NA
**Melanesia and West Polynesia**	Pre-Lapita	Pre-3500	Pre-1550 BCE	[9,10]
	Lapita	3500–2100	1550–150 BCE	[9,[11], [12], [13]]
	Post-Lapita	2100–450	150 BCE–1500 CE	[[11], [12], [13]]
	Protohistoric	450–250	1500–1700 CE	[13]
	Historic (contact)	Post-250	Post-1700 CE	[13]
**East Polynesia**	Early Prehistoric	Pre-550	Pre-1400 CE	[[14], [15], [16]]
	Late Prehistoric	550–250	1400–1700 CE	[[14], [15], [16]]
	Historic (contact)	Post-250	Post-1700 CE	[[14], [15], [16], [17]]
**Micronesia**	Pre-Latte	Pre-950	Pre-1000 CE	[18,19]
	Latte	950–250	1000–1700 CE	[18,19]
	Historic (contact)	Post-250	1700 CE	[18]

Laboratory (‘Lab name’), ‘Sample preparation’ and ‘Analysis’ data are provided for each isotopic measurement under fields specific to the different types of isotopic proxy: organic δ^13^C, δ^15^N, and δ^34^S; carbonate δ^13^C and δ^18^O; phosphate δ^18^O; and ^87^Sr/^86^Sr isotope analyses. For organic δ^13^C and relative uncertainty, whether the isotopic measurement (‰ relative to VPDB standard) refers to an AMS or IRMS analysis is flagged. Only IRMS measurements and uncertainties are provided for organic δ^15^N (‰ relative to AIR standard), and δ^34^S (‰ relative to VCDT standard). For human and non-human animal remains ‘Collagen yield’ is also provided, together with ‘Atomic C/N ratio’, and ‘%C’ and ‘%N’ content, whenever reported in the original publication. This data allows for the reliability of the measurements to be ascertained [7], and for data filtering to occur where necessary. ‘Atomic C/S ratio’ and ‘Atomic N/S ratio’ and ‘%S’ are also provided as measures of data reliability for δ^34^S measurements [8]. Carbonate δ^13^C (‰ relative to VPDB standard) and δ^18^O measurements and uncertainties and phosphate δ^18^O are also reported in the database. For both carbonate and phosphate δ^18^O, different fields register isotopic measurements (in ‰) whether reported in the original publication as relative to either the VPDB or VSMOW standard. ^87^Sr/^86^Sr measurements and uncertainties are also included in OAPID.

## 4 Experimental Design, Materials and Methods

Original publications and compilations containing carbon, nitrogen, oxygen, strontium and sulphur isotope measurements on human, non-human animals or plants from archaeological or palaeontological sites in Oceania were located using the online scientific search engine Google Scholar. Metadata information concerning the site, sample, context, chronology and isotopic measurements from these publications were extracted and recorded. The database was compiled using Excel. Literature published up to 1 Jan 2025 is included in the database.

## Limitations

The data compiled in the OAPID database is limited to published research into carbon, nitrogen, oxygen, strontium and sulphur isotope measurements on human, non-human animals or plants from archaeological or palaeontological sites in Oceania available on 1 Jan 2025. The database does not include isotopic research on contemporary samples.

There are large regional gaps in this data, especially for continental Australia (see Fig. 1). We hope that the publication of OAPID will allow researchers to identify key regional gaps in research and begin to remedy them.

## Ethics Statement

The authors have read and followed the ethical requirements for publication in *Data in Brief*. The OAPID database was compiled from already completed isotopic research. As such, the research published in this manuscript did not involve human subjects, animal experiments, or any data collected from social media platforms.

## CRediT Author Statement

**S.A. Florin:** Conceptualization, Investigation, Data curation, Validation, Writing –original draft; **A. Rogers:** Conceptualization, Investigation, Data curation, Validation, Writing –review & editing; **N.B. Lyall:** Investigation, Data curation, Writing –review & editing; **A. Crowther:** Conceptualization, Data curation, Writing –review & editing; **C. Cocozza:** Conceptualization, Validation, Writing –review & editing; **R. Fernandes:** Conceptualization, Writing –review & editing.

## Acknowledgments

The data was compiled and curated as part of the Pandora & IsoMemo initiatives supported by the Max Planck Society, PS&H research group, University of Warsaw, Masaryk University, and Eurasia3angle research group. Modelling infrastructure development was supported by DARIAH-PL and the Max Planck Society. SAF is supported by an Australian National University Futures Scheme 2.0, and AJR is supported through the Monsh University Faculty of Arts New Appointees grant.

## Declaration of Competing Interest

The authors declare that they have no known competing financial interests or personal relationships that could have appeared to influence the work reported in this paper.

## Data Availability

PandoraOceanian Archaeological and Palaeontological Isotope Database (Reference data).

## References

[1] Clarkson C., Jacobs Z., Marwick B., Fullagar R., Wallis L., Smith M., Roberts R.G., Hayes E., Lowe K., Carah X., Florin S.A., McNeil J., Cox D., Arnold L.J., Hua Q., Huntley J., Brand H.E.A., Manne T., Fairbairn A., Shulmeister J., Lyle L., Salinas M., Page M., Connell K., Park G., Norman K., Murphy T., Pardoe C. (2017). Human occupation of northern Australia by 65,000 years ago. Nature.

[2] Leavesley M.G., Bird M.I., Fifield L.K., Hausladen P.A., Santos G.M., di Tada M.L. (2002). Buang Merabak: early evidence for Human occupation in the Bismarck Archipelago, Papua New Guinea. Austral. Archaeol..

[3] Bunbury M.M.E., Petchey F., Bickler S.H. (2022). A new chronology for the Maori settlement of Aotearoa (NZ) and the potential role of climate change in demographic developments. Proceed. Nation. Acad. Sci. USA.

[4] Burley D., Weisler M.I., Zhao J.X. (2012). High precision u/th dating of first Polynesian settlement. PLoS. One.

[5] Petchey F., Spriggs M., Bedford S., Valentin F., Buckley H. (2014). Radiocarbon dating of burials from the Teouma Lapita cemetery, Efate, Vanuatu. J. Archaeol. Sci..

[6] Buikstra J.E., Ubelaker D.H. (1994). Standards for data collection from human skeletal remains: proceedings of a seminar at the Field Museum of Natural History organized by Jonathan Haas. Arkansas Archaeolog. Surv., Fayetville.

[7] Ambrose S.H. (1990). Preparation and characterization of bone and tooth collagen for isotopic analysis. J. Archaeol. Sci..

[8] Nehlich O., Richards M.P. (2009). Establishing collagen quality criteria for sulphur isotope analysis of archaeological bone collagen. Archaeol. Anthropol. Sci..

[9] Specht J., Fullagar R., Torrence R. (1991). What was the significance of Lapita pottery at Talasea?. Bullet. Indo-Pacific Prehist. Associat..

[10] Rieth T.M., Morrison A.E., Addison D.J. (2008). The temporal and spatial patterning of the initial settlement of Sāmoa. J. Island Coastal Archaeol..

[11] Summerhayes G.R., Gadu M.Z., Lin H.-M. (2010). 2009 International Symposium on Austronesian Studies, National Museum of Prehistory, Taitong.

[12] Summerhayes G.R., Allen J., Bedford S., Sand C., Connaughton S.P. (2007). Oceanic Explorations: Lapita and Western Pacific settlement.

[13] Cruz Berrocal M., Uriarte González A., Millerstrom S., Consuegra Rodríguez S., Pérez-Arias J., Ormeño S. (2014). Archaeological history of a Fijian island: moturiki, Lomaiviti Group. Asian Perspectives.

[14] McCoy M.D. (2007). A revised Late Holocene culture history for Moloka'i Island, Hawai'i. Radiocarbon..

[15] Dudgeon J.V. (2008).

[16] Kahn J.G. (2006). Society Islands (Central Eastern Polynesia) chronology: 11 radiocarbon dates for the late prehistoric expansion and proto-historic periods in the ‘Opunohu Valley, Mo'Orea. Radiocarbon..

[17] Mulrooney M., Ladefoged T.N., Stevenson C.M., Hanoa S. (2009). The myth of A.D. 1680: new evidence from Hanga Ho’onu, Rapa Nui (Easter Island),. Rapa Nui J..

[18] Carson M. (2012). An overview of *latte* period archaeology. Micronesia.

[19] Boyd B., Dega M., Moore D., Amesbury J.R. (2022). Archaeological research at the early pre-latte period site of San Roque on Saipan (ca. 1500–1100 BC). J. Isl. Coast. Archaeol..

[20] Rogers A.J., Florin S.A., Crowther A., Lyall N.B., Cocozza C., Fernandes R. (2026). A meta-analysis of stable isotope data from early Pacific Island colonisation to complex chiefdoms. OSF Preprint.

